# Design, Validation, and Fabrication of a Tailored Electrochemical Reactor Using 3D Printing for Studies of Commercial Boron-Doped Diamond Electrodes

**DOI:** 10.1021/acs.iecr.3c03123

**Published:** 2024-03-25

**Authors:** Lais Vernasqui, Miguel A. Montiel, Neidenêi Gomes Ferreira, Pablo Cañizares, Manuel A. Rodrigo

**Affiliations:** † Department of Chemical Engineering, Faculty of Chemical Sciences & Technologies, University of Castilla-La Mancha, Campus Universitario s/n, 13071 Ciudad Real, Spain; ‡ National Institute for Research Space, Av. dos Astronautas, 1.758-Jardim da Granja, São José dos Campos, São Paulo 12227-010, Brazil

## Abstract

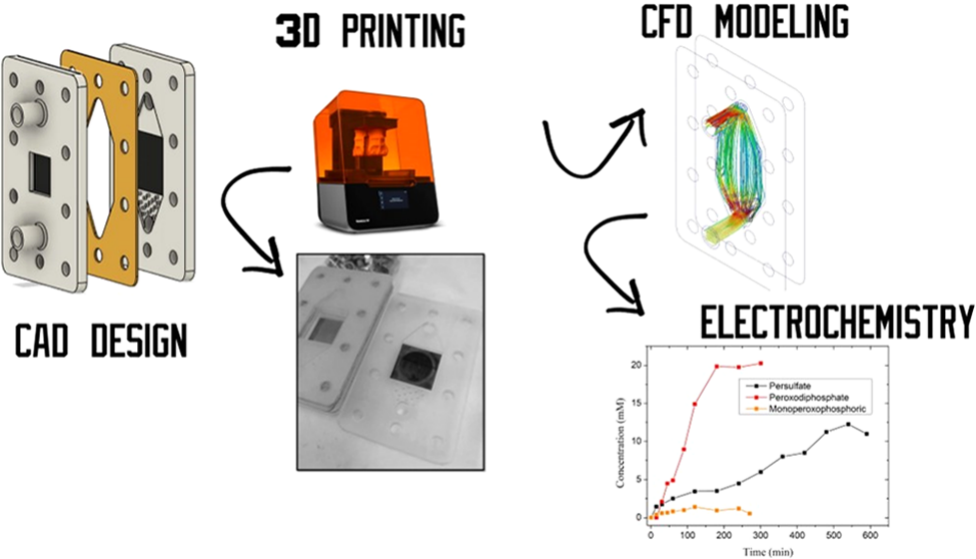

Boron-doped diamond (BDD) electrodes are the most effective and resistant electrodic materials to perform advanced oxidation processes. Having a reactor that can provide adequate hydrodynamic conditions is mandatory to use these electrodes effectively. In this work, the diamond anode electrochemical reactor (E3L-DAER) is designed to fulfill this necessity. Several features are included to improve its efficiency, like conic inlet/outlet, flow enhancers, and a reduced interelectrode gap. The fluid dynamic validation has been performed using computer fluid dynamics (CFD) calculations, residence time distribution (RDT) curves, and mass transfer analysis. The reactor has been made using a three-dimensional (3D) printing stereolithography (SLA) technique, which allows us to build chemical-resistant reactors with nonstandard and tailored features in a cheap and fast way. The obtained results demonstrate that the designed reactor has the required fluid dynamics properties to perform reliable BDD electrode studies and applications. Finally, a BDD electrode was used to test the production of different oxidants such as persulfate, peroxophosphate, and chlorine-derived species.

## 1 Introduction

With the rapid and impressive development of three-dimensional (3D) printing technology in recent years, electrochemical technology researchers are being motivated to make a rapid shift toward the development of more efficient electrochemical cells.^1,2^ Research in electrochemical reactors involves addressing different properties such as the influence of mass transport.^3−5^ Regarding the application of electrochemical technologies to water treatment, and more specifically, the electrochemical production of oxidants, most of the research carried out in the last two decades has focused on the development and characterization of electrodes with high efficiencies in the reaction of interest as well as a long useful life. In this context, boron-doped diamond (BDD) electrodes have been demonstrated to be a suitable solution to many environmental problems and also for electroanalysis of different molecules due to their unique properties, such as wide oxidation potential windows, low background current, corrosion resistance, and electrochemical stability.^6,7^ Furthermore, the exceptionally efficient generation of hydroxyl radicals plays a critical role in promoting the creation of various oxidants within the bulk electrolyte during the electrolysis process. This includes the generation of hydrogen peroxide, ozone, and, depending on the electrolyte composition, other species such as peroxosulfates, peroxocarbonates, peroxophosphates, and more.^8−12^

However, in the last five years, progress in the development of this technology slowed down, and no relevant challenges were identified except the search for synergistic combinations between electrochemical technology and other technologies looking for the activation of oxidants during the electrolytic process, especially with photochemical and sonochemical technologies.^13−16^ Also, the search for new applications and the expansion of the possibilities of treating liquid to gaseous waste through electrochemically assisted reactive absorption processes was also set as a new objective, something that has been successfully addressed.^17−19^

Designing electrochemical cells requires careful evaluation of many concurrent processes that are not normally faced during all these studies carried out in the last two decades. Minimizing the gap between electrodes can minimize the cell voltage and, consequently, the energy consumption of the electrolytic process. Efficient gas evacuation improves the performance by using the electrode surface more efficiently. The distribution of the flow inside the cell helps to attain a uniform current distribution, which may impact reaching higher efficiencies, even more than the electrocatalytic characteristics of the electrodes. Also, the current feeder can have a very important impact on this uniform current distribution, which is necessary to obtain highly effective electrochemical processes. These are some inputs that support the need to change the research paradigm in electrochemical technology and achieve faster progress in the development of electrochemical cells, paying more attention to the reactor than to reaction engineering.^20−23^

Up to now, the design of electrochemical cells has involved the extensive use of computer fluid dynamics (CFD) modeling.^24−26^ This methodology was highly time-consuming, simulation results did not always fit the observed experimental behavior, and in fact, conclusions were not always easy to extrapolate to reality. This fact had a direct impact on the market availability of electrochemical cells with a very limited number of options. Consequently, most of the research works only evaluated very simple in-house-made flow cells or the cells of three to four manufacturers.^27,28^ The fast and cheap production of cells with 3D printing technology is changing the paradigm, and now, it is possible to conceptualize, design, and manufacture cells in a very short time, saving a lot of time and having a real view of their performance with details, which is difficult to obtain using only CFD modeling.^29,30^

This work has been focused on the design of a new cell capable of exploiting the high capacities of diamond coatings in order to reach extremely high efficiencies in processes in which this type of electrode has demonstrated a super performance compared to conventional electrodes. Prototyping and testing are aimed at reaching an electrochemical cell in which the good performance of the diamond electrode is not limited by reactor weaknesses. The results conclude with the prototyping of the diamond anode electrochemical reactor (E3L-DAER), a new cell whose high performance was tested in the production of peroxo species and chlorate.

## 2 Materials and Methods

### 2.1 Cell Desing Criteria

The objective of the internal development and production of the reactor aims to overcome one of the main problems of the generation of oxidants with BDD electrodes, namely, the generation of scavenger species and its further reaction in the homogeneous phase. To achieve this goal, efficient mass transport is imperative to evacuate the reactants generated over the surface in a rapid way, either gas or solvated. Also, energy effectiveness and cost reduction are considered in the design. For this reason, the reactor design must address several challenges. First, it is necessary to avoid additional components due to the harsh oxidizing conditions that BDD oxidation experiments produce, which require refractory materials that are often expensive and difficult to manufacture or prevent their reaction with the generated oxidants. Second, the oxidation processes on BDD surfaces necessitate a high overvoltage and lead to significant gas evolution at the cathode and anode (water hydrolysis). This requires a reactor that can effectively evacuate the gas while benefiting from this gas evolution with a high linear speed and homogeneous distribution. Finally, achieving a low cell voltage and minimizing pump power can help to reduce energy consumption and therefore facilitate future system scaling. Computer-aided design (CAD), computational fluid dynamics (CFD) simulation, and 3D printing present an opportunity to rapidly optimize reactor design to overcome these challenges.

Overall, CAD design, CFD simulation, and the 3D printing process can be described as cost-efficient. The materials used are affordable, manufacturing costs are low, and the process time is significantly reduced, as it does not rely on external designers or manufacturers. Throughout the discussion of the results, various tests and studies have been conducted, including the calculation of mass transport coefficients, residence time distribution (RTD) curves, and CFD simulation. These analyses were performed to evaluate and confirm that the reactor design is optimal for the intended application. Moreover, through CFD calculations, other reactor geometries are included to discuss the effect of the key features of the E3L-DAER reactor.

Considering that, the electrochemical cell, referred to as E3L-DAER, possesses a filter-press configuration and has been meticulously designed utilizing Fusion 360 software. In essence, the design comprises two parallel polymer plates measuring 100 mm × 65 mm, which house two electrode sockets measuring 2.5 cm × 2.5 cm each. These sockets are connected to the inlet and outlet via a conical-shaped zone to facilitate optimal liquid distribution and efficient evacuation of the generated gas. Furthermore, the design incorporates additional flow enhancers in the inlet area to favor a uniform distribution of the liquid across the electrode surface. The separation distance between the electrodes is maintained at 3 mm to ensure a high linear speed even at low flow rates, thereby positively impacting mass transport. Although this work employs a one-compartment configuration, the cell is capable of operating in double-compartment mode by incorporating a membrane.

### 2.2 CFD Calculations

CFD simulation was conducted using Autodesk CFD software. A K-epsilon turbulence model was selected to comprehensively assess the impact of turbulence generated by the flow enhancers located at the cell’s inlet. An adaptive mesh setting was applied, resulting in three cycles of 100 iterations each until mesh independence of over 95% was achieved for both pressure and velocity magnitude. The performed procedure yielded a mesh comprising 57,390 nodes and 247,400 elements. The boundary conditions were set as a flat velocity (mm s^–1^) profile for the fluid at the cell’s inlet while maintaining a uniform gauge pressure of 0 at the outlet. Four distinct cases were examined and introduced as boundary conditions, where the gauge pressure at the outlet remained constant at 0, while the inlet fluid velocity varied according to the different flow rates investigated in this study (15, 30, 50, and 65 L h^–1^).

### 2.3 3D Printing of the E3L-DAER Electrochemical Reactor

Stereolithography, a 3D printing technique, was employed by using a Form 3 machine procured from Formlabs. The chosen material is a translucent acrylic-based resin known as Clear Resin V4, which was also acquired from Formlabs. The material was selected due to its resistance to acidic and alkaline media and favorable mechanical properties, enabling sufficient force application for reactor closure. During the 3D printing process, a thin layer of the resin is applied and subsequently exposed to a lens-focused ultraviolet (UV) laser, causing photopolymerization of the resin layer by layer. This precise method results in minimal material waste compared with alternative manufacturing techniques such as computer numerical control (CNC) machining. Following the printing of the cell pieces, a thermal UV treatment is necessary to fully optimize the mechanical and chemical properties of the material. The specific treatment conditions are specified by the supplier, being 15 min at 60 °C. Subsequently, the BDD (boron-doped diamond, anode) and SS (stainless steel, cathode) electrodes are affixed to the printed parts using epoxy resin, and electrical contact is established through cold welding on the electrode’s backside. The stability of the polymerized resin material was evaluated by immersing a small resin sample in 1 M H_2_SO_4_ and 0.1 M KOH for a period of 4 weeks, revealing no notable changes in color, weight, or hardness.

### 2.4 Residence Time Distribution (RTD) Curves and Mass Transfer Coefficient Calculation

Experimental RTD curves were performed using an ionic tracer.^31^ A 2 mL dose of 5 M NaCl was injected at the reactor inlet, while conductivity was measured at the outlet for different times and flow rates. Also, experimental data were fitted to the dispersion RTD model shown in eq 1, where *E*(*t*) is the conductivity measured divided by the integral of the conductivity curve with respect to time, θ is the mean hydraulic residence time, *D*_L_/*vL* is the Péclet number, and *t* is time.
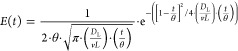
1

The mass transfer coefficients in the E3L-DEAR reactor were determined using the limiting current technique.^32,33^ The electrolyte solution utilized in the experiment consisted of 0.50 M Na_2_CO_3_ as the supporting electrolyte, with 0.05 M K_4_Fe(CN)_6_ as the compound to be anodically oxidized and 0.10 M K_3_Fe(CN)_6_ as the compound to be catholically reduced. The concentration of ferricyanide was intentionally maintained at twice the concentration of ferrocyanide to ensure that the oxidation of ferrocyanide is limited to the anode. To prevent the decomposition of the ferro/ferricyanide solution, the entire system, including pipes and the electrolyte storage tank, was isolated from UV light. The electrolysis process was driven by a power supply capable of providing a voltage range of 0–30 V and a current range of 0–10 A. This test finds the conditions in which the cell was operating under mass transfer control. In these conditions, the mass transfer coefficient on the surface of the anode can be correlated with the limiting current using eq 2, where *I*_lim_ is constant at the potential values where the reaction is controlled by mass transfer, *F* is the Faraday constant, *n* is the number of electrons exchanged, *C*_bulk_ is the concentration of K_4_Fe(CN)_6_, and *A* is the geometric electrode area.
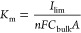
2

### 2.5 Electrochemical Synthesis of Different Oxidant Species

The BDD electrode used in this work was purchased from Adamant Technologies (Switzerland), and its properties are described in Table 1. The film characteristics shown in Table 1 were provided by Adamant Technologies, and this electrode was previously used (same type of electrode but a different sample) to evaluate the electrochemical production of perphosphate.^27^ As the cathode, stainless steel 314 was used.

**Table 1:** Characteristics of the Conductive Diamond Electrode Used in This Work

	conductive diamond layer			*p*-Si substrate	
commercial reference	boron content (ppm)	sp^3^/sp^2^ ratio	BDD layer thickness (μm)	Si resistivity (mΩ·cm)	Si surface roughness (μm)
WD 908–5	2500	68	1.15	100	<0.1

Different oxidant species were evaluated by the application of the BDD electrode during electrolysis in a flow system: persulfate, monoperoxophosphoric acid, peroxodiphosphate, and oxidation chlorine products. Besides the electrochemical cell, the flow system used in all cases was composed of a jacket reservoir that allows temperature control, a peristaltic pump (model 7519–40 from Masterflex) with flow control, and a power source (model ES030–10 (30 V/10 A) from Delta Elektronica). In all cases, the flow and temperature were kept constant at 50 L h^–1^ and 25 °C, respectively, and two different current intensities were tested, i.e., 25 and 300 mA cm^–2^.

For the evaluation of persulfate, electrolysis was conducted in 1 M H_2_SO_4_, with the pH adjusted to 7. In the peroxophosphate production, 1 M H_3_PO_4_ was used with the pH adjusted to 1 and 12, depending on the experiment. Iodometric titration was used to quantify the oxidant formation.^34^ The formation of oxidized chlorine products was conducted by the electrolysis of 0.5 M NaCl, and they were quantified by ion chromatography (Metrohm 930 Compact IC Flex fitted with a conductivity detector and a Metrosep A Supp 7 column as stationary phase). The mobile phase was an 85:15 v/v 3.6 mmol L^–1^ sodium bicarbonate/acetone solution flowing at 0.80 cm^3^ min^–1^, the oven temperature was 45 °C, and the injection volume was 20 μL.

### 2.6 Chemicals and Reagents

All chemicals were purchased from Merck (Germany) NaCl ≥ 99%, H_2_SO_4_ ≥ 98%, H_3_PO_4_ ≥ 85%, Na_2_S_2_O_3_ ≥ 99%, KI ≥ 99%, and KOH ≥ 98%.

## 3 Results and Discussion

### 3.1 Fluid Dynamics Study and Validation

**Figure 1:**
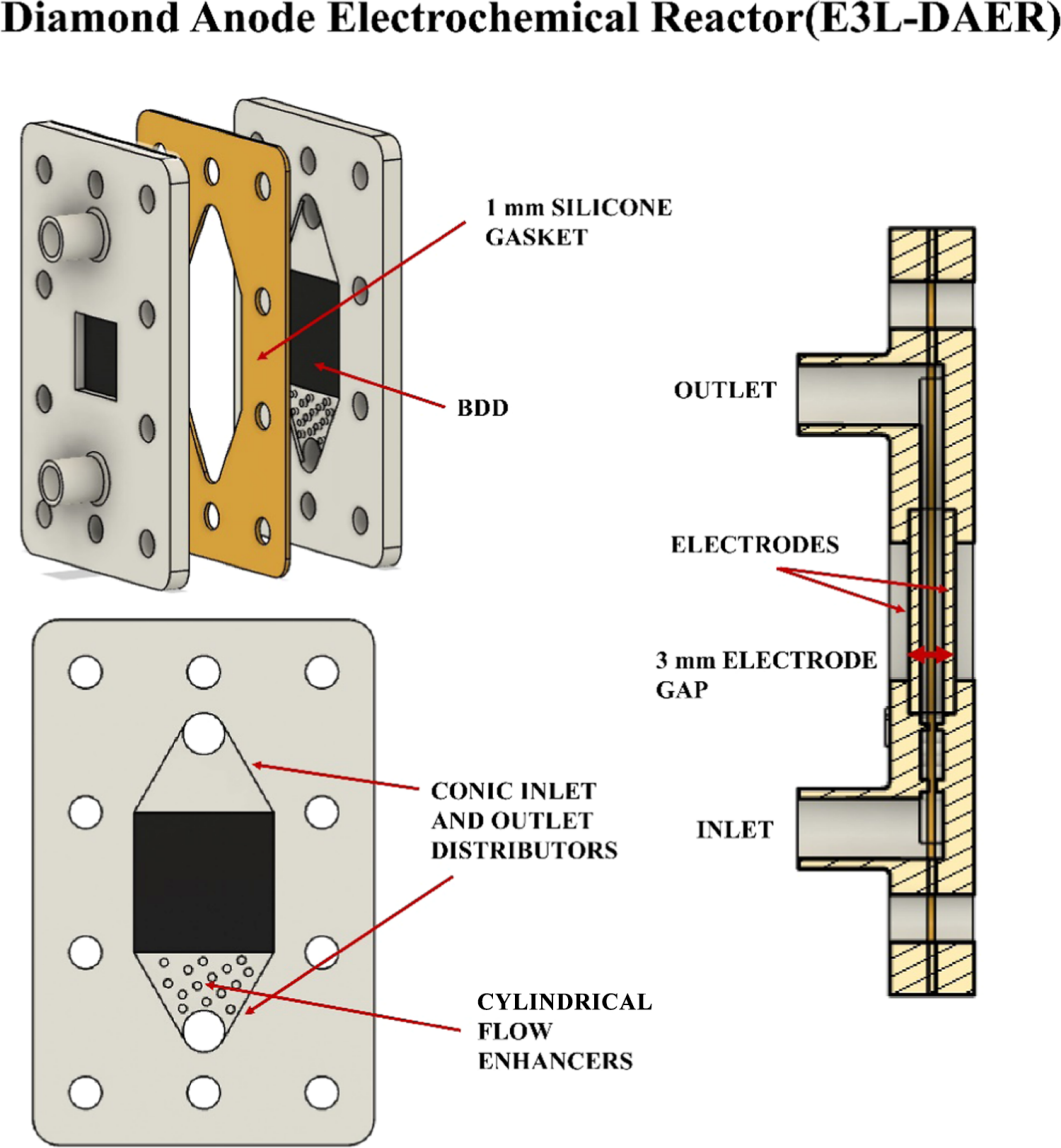
Scheme of the E3L-DAER electrochemical reactor. Left: isometric and *XZ* plane views; right: *XZ* section plane.

Figure 1 shows the scheme of the E3L-DAER electrochemical cell, pointing out mechanical characteristics worth to be highlighted. Thus, the use of an electrochemical filter-press reactor usually demands a turbulence promoter as the primary additional component. This element, typically made of plastic, enhances mass transport and improves the distribution of the fluid in the *XY* and *YZ* planes. To eliminate the need for a turbulence promoter, various characteristics are integrated into the reactor design (Figure 1). First, the interelectrode gap is reduced to 3 mm to maximize mass transport. Second, a cone-shaped inlet with circular flow enhancers is incorporated into the design to promote a homogeneous flow distribution in a similar way as was reported by Márquez-Montes et al. and López-Maldonado et al.^4,35^ Regarding the gas evacuation, the combination of the small interelectrode gap and the conical-shaped outlet of the cell contributes to the efficient evacuation of gas by increasing the fluid velocity and avoiding the formation of dead zones. Finally, the two main energy drivers in this process are the cell voltage (assuming that current is a constant parameter) and the pumping requirement. Once again, the design features introduced will contribute to reducing the energy demand in both pumping energy and particularly the cell voltage.

**Figure 2:**
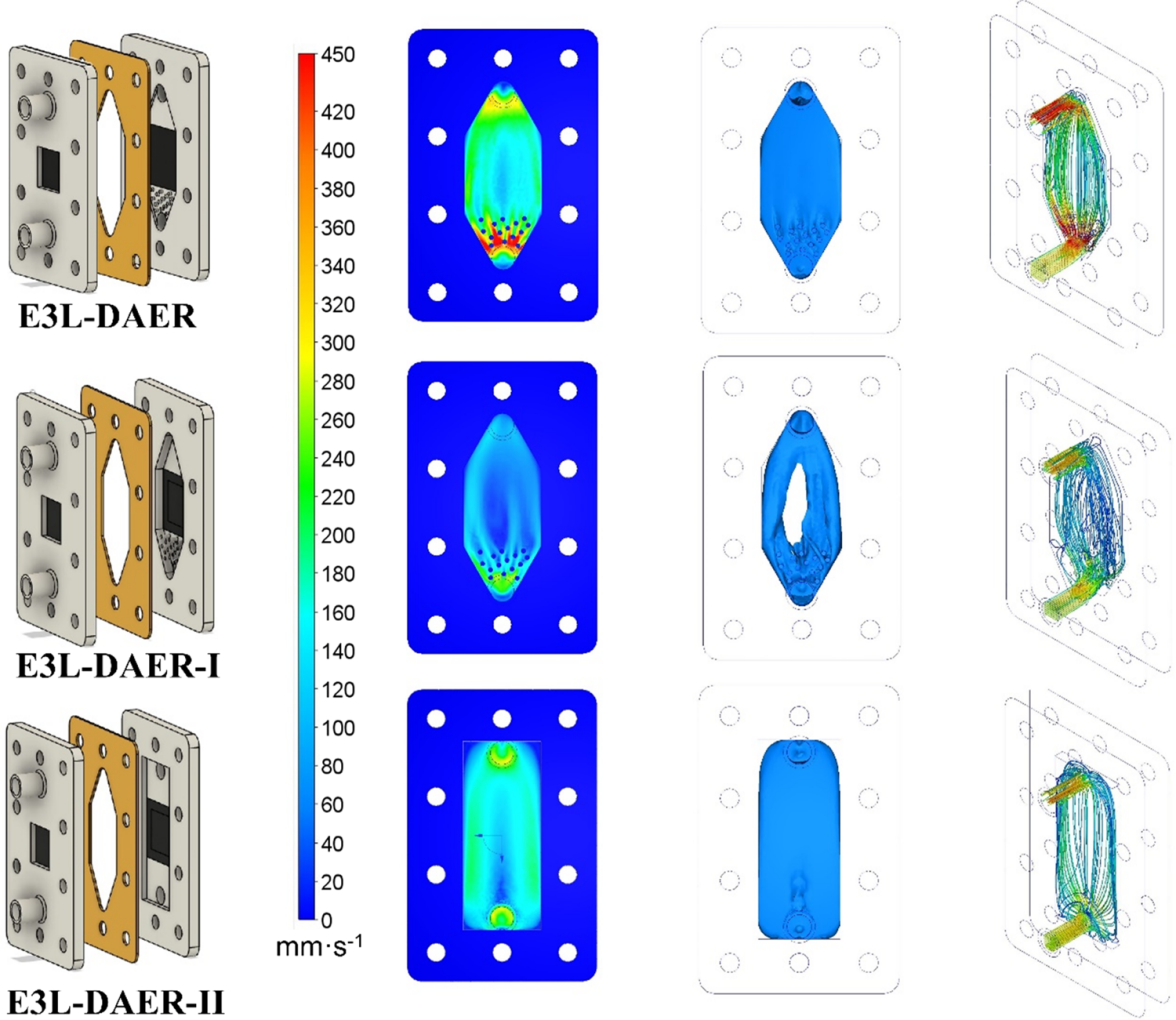
From left to right, isometric views, CFD calculations of fluid linear velocity (mm s^–1^) in the *XZ* plane, CFD calculated isosurfaces at 60 mm s^–1^ fluid speed, and calculated fluid paths of the three reactor variations studied at 50 L h^–1^.

CFD simulation was used to determine the effectiveness and convenience of the key features incorporated into the E3L-DAER reactor. For this reason, two additional alternative designs were proposed. In the E3L-DAER-I reactor, the electrode gap is set to 10 mm instead of the 3 mm of the E3L-DAER reactor. 10 mm is closer to the typical value used in regular filter-press reactors.^36,37^ Second, in the E3L-DAER-II reactor, both the cylindrical flow enhancers and cone-shaped inlet and outlet are removed. The schemes of these two E3L-DAER modifications can be seen on the left part of Figure 2. To fully compare the three proposed designs, from left to right in Figure 2, the linear velocity profile in the *XZ* plane, isosurface representations at 60 mm s^–1^, and calculated fluid tracers at a 50 L h^–1^ flow rate are used. Regarding the linear velocity profile, when the electrode gap is increased to 10 mm (In E3L-DAER-I), the linear speed drops dramatically, while several dead zones (spots with low lineal velocity marked in dark blue) appear over the electrode. This may lead to a poor mass transfer and poor evacuation of the products formed during the reaction, either gas or liquid. In E3L-DAER-II, a decrease in linear speed and a more uneven distribution are observed, with a slower flow in the central part of the cell and dead zones in the upper corners, which can produce gas pockets during operation. The results observed in the linear velocity *XZ* planes are confirmed by the isosurfaces at 60 mm s^–1^, where color voids are observed, indicating lower linear velocities than 60 mm s^–1^. The calculated profiles show in blue the surface at which the linear velocity of the fluid is at least 60 mm s^–1^. This value was selected because the distance between the inlet and the outlet was 60 mm; for instance, if all the reactor volume is covered by the isosurface (blue surface like in E3L DAER geometry), the residence time will be aproximatelly 1 s or less. Finally, in the right part of Figure 2, the same number of particle tracers are introduced in the three cell models. The path described by the particle is represented on the same color scale as linear velocity. Both by the velocity reached and a more homogeneous distribution of the lines, the E3L-DAER reactor shows a better performance to the application that the reactor is intended for. Therefore, the E3L-DAER reactor geometry is preferred over E3L-DAER I and II because of the avoidance of the formation of dead zones, which can lead to poor electrode performance or active area loss due to gas pocket formation near the electrode surface.

**Figure 3:**
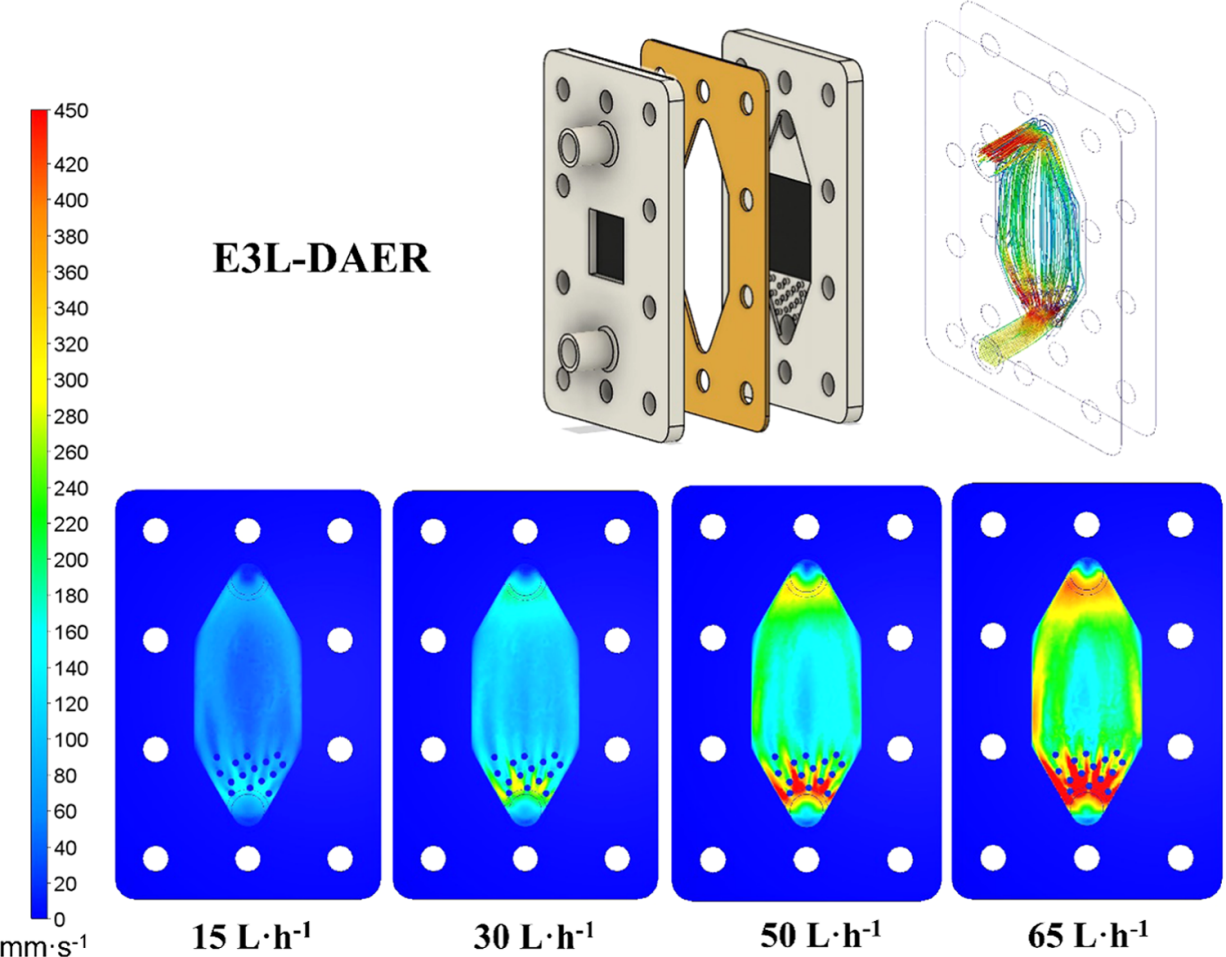
CFD calculations of the fluid linear velocity (*YZ* plane) at four different flow rates.

Once it was confirmed that the E3L-DAER reactor was the optimal geometrical design choice for the test of BDD electrodes, it was printed using the SLA technique. The reactor material was carefully selected and tested, with Clear Formlabs Resin V4 being the chosen one. It was tested under acidic and oxidative stress conditions with no appreciable midterm (4 weeks) damage. The next step was to evaluate by both CFD calculations and experimental methods the adequate liquid flow to perform experiments with the E3L-DAER reactor. In the first place, CFD calculations were done at four different flows (15, 30, 50, and 65 L h^–1^), which are depicted in Figure 3. The target is to have a linear velocity over 60 mm s^–1^ along all of the reactors and achieve also higher velocities at the inlet/outlet to fulfill a good fluid distribution and gas evacuation. In the first place, at 15 L h^–1^, the target fluid velocity is not reached, while at 30 L h^–1^, despite reaching 60 mm s^–1^ in a major part of the reactor, the velocity at the outlet and the side parts of the reactor is not enough to ensure a fast and reliable gas evacuation. Note that this feature is crucial because of the narrow interelectrode gap of 3 mm. If any gas pocket or big bubble is formed, it will considerably diminish the reactor function. On the other hand, at 50 L h^–1^, while maintaining the minimum 60 mm s^–1^ velocity across the reactor, the side, inlet, and outlet parts are at least at 200 mm s^–1^. This velocity guarantees not only a proper gas evacuation but also a short residence time of the generated oxidant products inside the reactor, minimizing the possible side reactions at the homogeneous phase. Pushing to 65 L h^–1^ further increases the velocity but does not represent a quality gap from 50 L·h, and other problems may occur like a higher pumping consumption or pressure drop.

**Figure 4:**
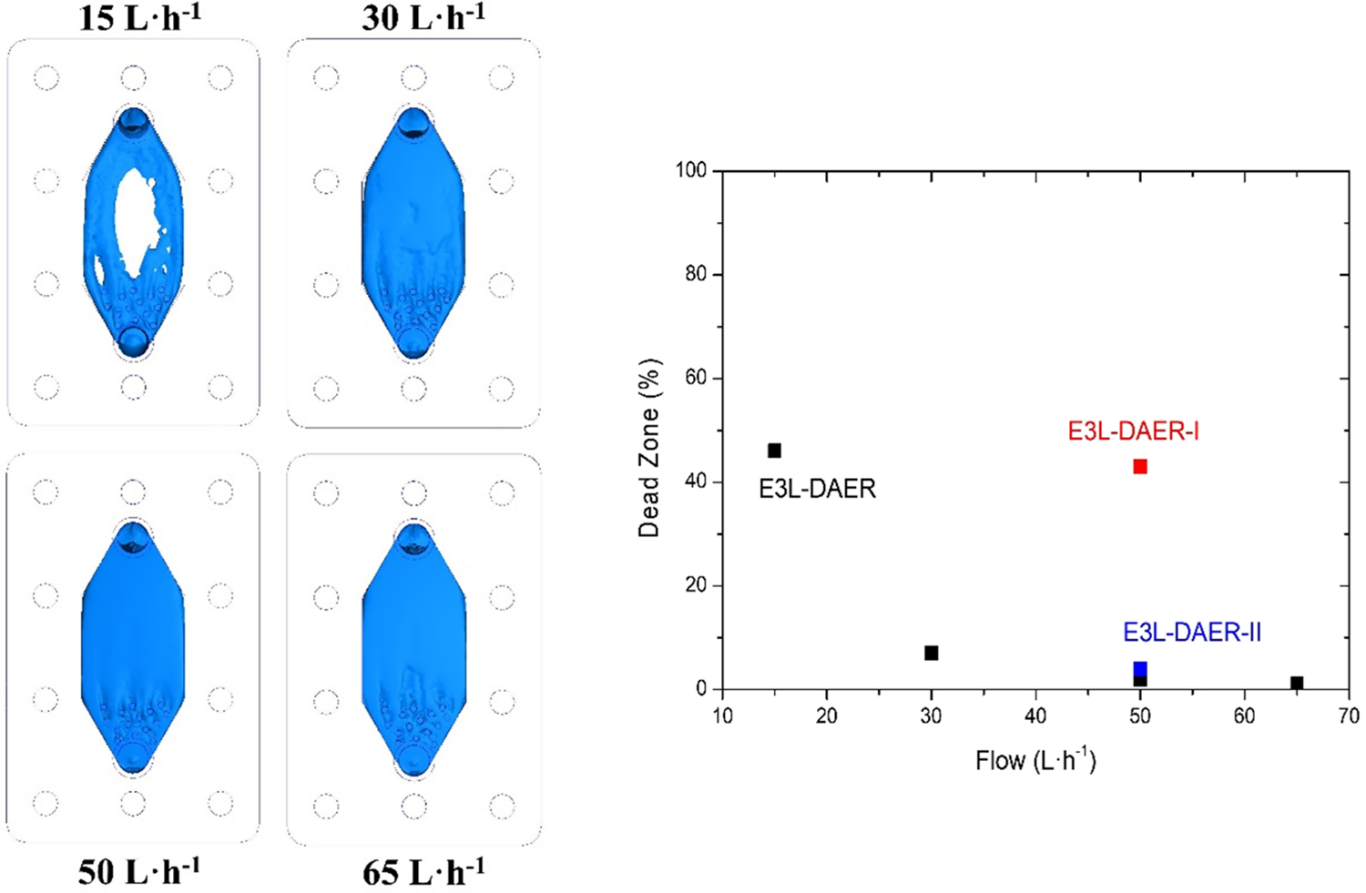
Isosurfaces in which the fluid velocity is at least 60 mm s^–1^ (left) and the % of dead zones at the electrode surface (right graph) at different flow rates. For 50 L h^–1^, the three studied reactors are represented in black (E3L-DAER), red (E3L-DAER-I), and blue (E3L-DAER-II).

Isosurfaces at 60 mm s^–1^ shown in Figure 4(left) demonstrate that over 30 L h^–1^, the fluid velocity surpasses the fixed value, while at 15 L h^–1^, 46% of the electrode surface is a dead zone. However, when a deeper colorimetric analysis is performed, some differences are seen between 30 and 50 L h^–1^. The right graph of Figure 4 shows the % of dead zones versus the flow rate; also, the other studied reactor variations are represented. While at 30 L h^–1^, dead zones represent 7% of the electrode surface, at 50 L h^–1^, it is only 2%. When increasing even more the flow to 65 L h^–1^, the % drops to 1. For this reason, the 50 L h^–1^ flow is selected as optimal for the reactor operation because the difference from 30 L h^–1^ is appreciable, but the increase of power to reach 65 L h^–1^ does not correspond with a high advantage in hydrodynamic conditions. At the same time, the other proposed reactor geometries presented worse values of dead zones at 50 L h^–1^, namely, 43 and 4% for E3L-DAER-I and E3L-DAER-II, respectively.

**Figure 5:**
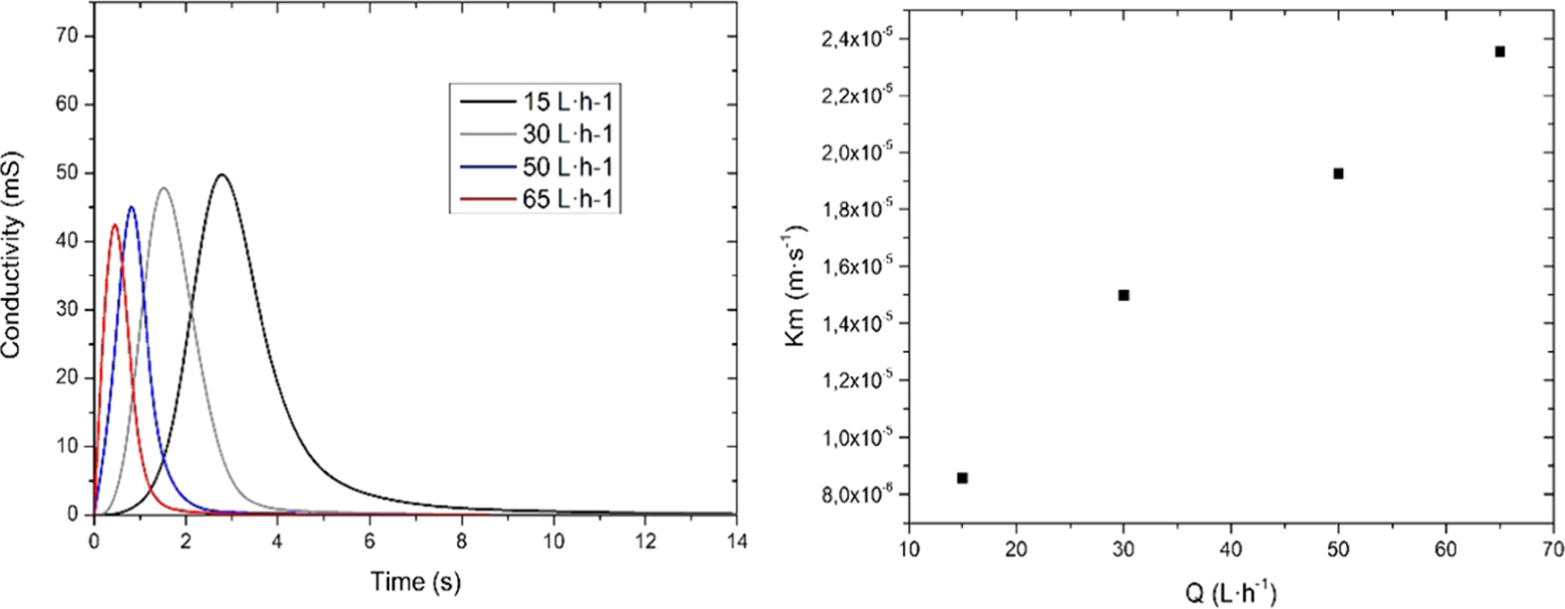
RTD of the E3L-DAER reactor (left) and determination of the mass constant (right) for four different flow rates.

Following fluid dynamics analysis of the reactor E3L-DAER, experimental tests were done. In the first place, RTD curves featuring conductivity versus time are shown in the left graph of Figure 5. The same four flow rates were analyzed by introducing an ionic tracer and then measuring the conductivity at the reactor outlet. As can be seen, when the flow is increased, the time that the tracer is inside the reactor descends. To better evaluate the residence time (θ) and the Péclet module (*P*_eL_), the curves are fitted to a model in Figure S1, and the results are plotted in Table 2. The residence time drops dramatically from 2.8 to 0.43 s when increasing the flow from 15 to 65 L h^–1^, while, surprisingly, *P*_eL_ increases. *P*_eL_ gives an idea of the quality of the mixing inside the reactor. When the *P*_eL_ module is close to 0, there is good mixing inside the reactor; this indicates that no dead zones are present. In the E3L-DAER case, and following the CFD calculations, a monotone dropping in *P*_eL_ was expected when increasing the flow; however, the results obtained with the conductivity experiments indicate that at high flows, and especially at 65 L h^–1^, this value rises. The reason is the possible formation of swirls that the CFD calculations were not capable of simulating. In conclusion and reminding the idea of having a flow pass through the cell of less than 1 s, the 50 L h^–1^ flow presented a 0.75 s θ without a significant *P*_eL_ increase, being confirmed as an excellent candidate for the use of the reactor operation.

**Table 2:** Values of Residence Time (θ), Péclet Module (*P*_eL_), and Fitting Error of the Modeled Curve (*r*^2^) for Different Flow Rates

flow rate (L min^–1^)	θ/s^–1^	*P*_eL_	*r*^2^
15	2.8	0.030	0.96
30	1.5	0.045	0.98
50	0.75	0.055	0.99
65	0.43	0.110	0.98

To estimate the transport of species inside the reactor, current *E*–*I* curves were made for the studied flow rates. Inside the reactor, the oxidation of ferrocyanide takes place at the BDD anode, reaching a current plateau that indicates that the process is controlled by mass transfer. Current values in that plateau are used to calculate mass transport coefficients (*K*_m_ in m s^–1^) and are further plotted in Figure 5 (right) versus the flow rate. The values obtained range from 0.8 to 2.35 × 10^–5^ m s^–1^ (15 to 65 L h^–1^ flow), which are in the typical range of a plane-parallel electrochemical reactor using electrodes without rugosity or 3D architecture.^38,39^ The value at 50 L h^–1^ is almost 2 × 10^–5^ m s^–1^, a value enough to perform degradation experiments if needed. Given the θ values obtained and the good velocity profile distribution, it was expected to have higher mass transport results; however, taking into account the design purpose of the reactor, it can be an advantage. If the mass transport is too high (range of 1 × 10^–4^), the transport species from the bulk to the surface and vice versa may favor the interaction between the scavengers generated, for example, at the cathode or even the cathodic degradation of the oxidant species generated. For this reason, having a moderated mass transport constant, but at the same time a high fluid velocity at the electrode surface and low θ, is the ideal scenario to evacuate the oxidant species generated at the electrode surface and minimize the action of both scavengers and cathodic degradation, that are well-known mechanisms of oxidant degradation species.^40−42^

To conclude with the fluid dynamics and mass transport analysis of the reactor, several features were implemented to the reactor to achieve: (i) low residence time of the oxidant species generated, (ii) no necessity of a turbulence promoter, (iii) good gas evacuation, and (iv) low cost. It has been demonstrated, both with CFD calculations and experimentally, that at 50 L h^–1^, all these conditions are fulfilled. Regarding the last point, the reactor cost in materials and operation time of the equipment used is 50 euros/54 dollars, a value that is far cheaper than the commercial units available or even buying the 3D print to a supplier. Moreover, the fabrication time is 24 h, thus allowing us to optimize the time for the validation of the desired reaction on BDD electrodes. Finally, the size of the socket bearing the BDD electrode is adaptable without appreciable modifications in the fluid dynamics properties if the specific features (conic inlet–outlet, flow enhancers, and interelectrode gap) are kept.

### 3.2 Electrochemical Validation

The electrochemical validation of the designed reactor was done using a BDD electrode, whose characteristics are shown in the experimental section. The main purpose of this validation is to assess the efficient production of different oxidant species using different electrolyte solutions. Four solutions were used, 1 M H_2_SO_4_, whose main oxidation products are persulfate species, 1 M H_3_PO_4_ adjusted to pH 12, whose main reaction product is peroxodiphospate, 1 M H_3_PO_4_ adjusted to pH 1, giving peroxomonophosphoric acid, and 0.5 M NaCl, where several oxidation species can be produced (hypochlorite, chlorate, and perchlorate). Regarding the production of these oxidant species, they can follow two different paths, the indirect oxidation by the formed OH· species at the electrode surface and the direct oxidation at the electrode surface.^41^

In more detail and in the case of persulfate, the direct and indirect formation is according to eqs 3–5.^28^
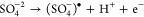
3
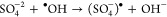
4
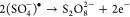
5

In the case of peroxodiphosphate, it is according to eqs 6–8.^27^
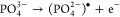
6
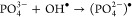
7
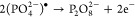
8

Regarding the chlorine species formation, it begins with hypochlorite production by the eq 9 pathway. In sequence, the chlorate (eq 10) and perchlorate (eqs 11 and 12) can be formed.^43^
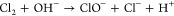
9
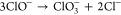
10
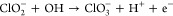
11
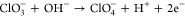
12

**Figure 6:**
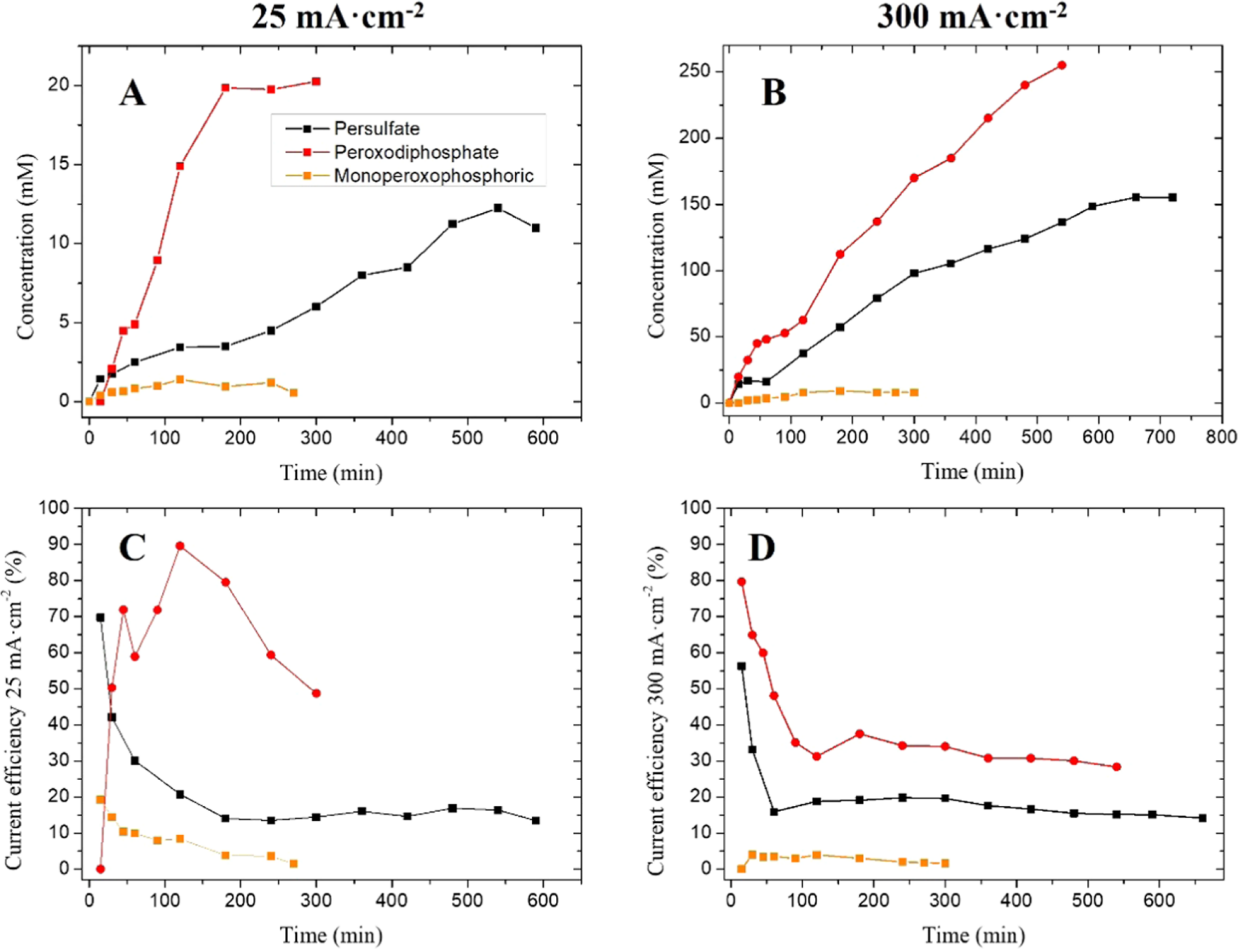
Oxidants produced in different electrolyte solutions: 1 M sulfuric acid (black), 12 M sodium phosphate (red), and 1 M phosphoric acid at pH 1 (orange). Production at 25 mA cm^–2^ (A) and its current efficiency (C), and production at 300 mA cm^–2^ (B) and its current efficiency (D).

As can be seen in Figure 6, the E3L-DAER reactor operates at two different current densities, 25 and 300 mA cm^–2^, to evaluate the impact of this parameter on the production and the current efficiency. Perphosphate species production is highly promoted at pH 12, as can be seen in Figure 6A,C, and is coincident with the previously reported data by Cañizares et al.^27^ However, current efficiencies of 25 and 300 mA cm^–2^ are superior to those reported by these authors using the same electrode, probably due to the highly favorable hydrodynamic conditions promoted by the E3L-DAER reactor. The efficiencies obtained at pH 12 and 25 mA cm^–2^ reach 90% at 120 min and 50% at 300 min, while those at pH 12 and 300 mA cm^–2^ are up to 50% at 90 min and over 30% at 480 min (Figure 6C,D). The local optimum efficiency at 120 min is explained because the concentration of perphosphate reaches a plateau at 120 min. In this plateau, the formation rate and decomposition (due to cathode decomposition and scavenger effect) rate of perphospate are equal. However, as current is still being applied without further perphosphate concentration increase, the same current efficiency decreases. Regarding the persulfate species generation, the efficiencies obtained at 25 and 300 mA cm^–2^ are similar and up to 15% at the steady-state concentration (600 min). This different behavior with the perphosphate species can be explained because in a single compartment and steady state, the production of persulfate species is strongly influenced by the decomposition rate at the cathode.^44^

**Figure 7:**
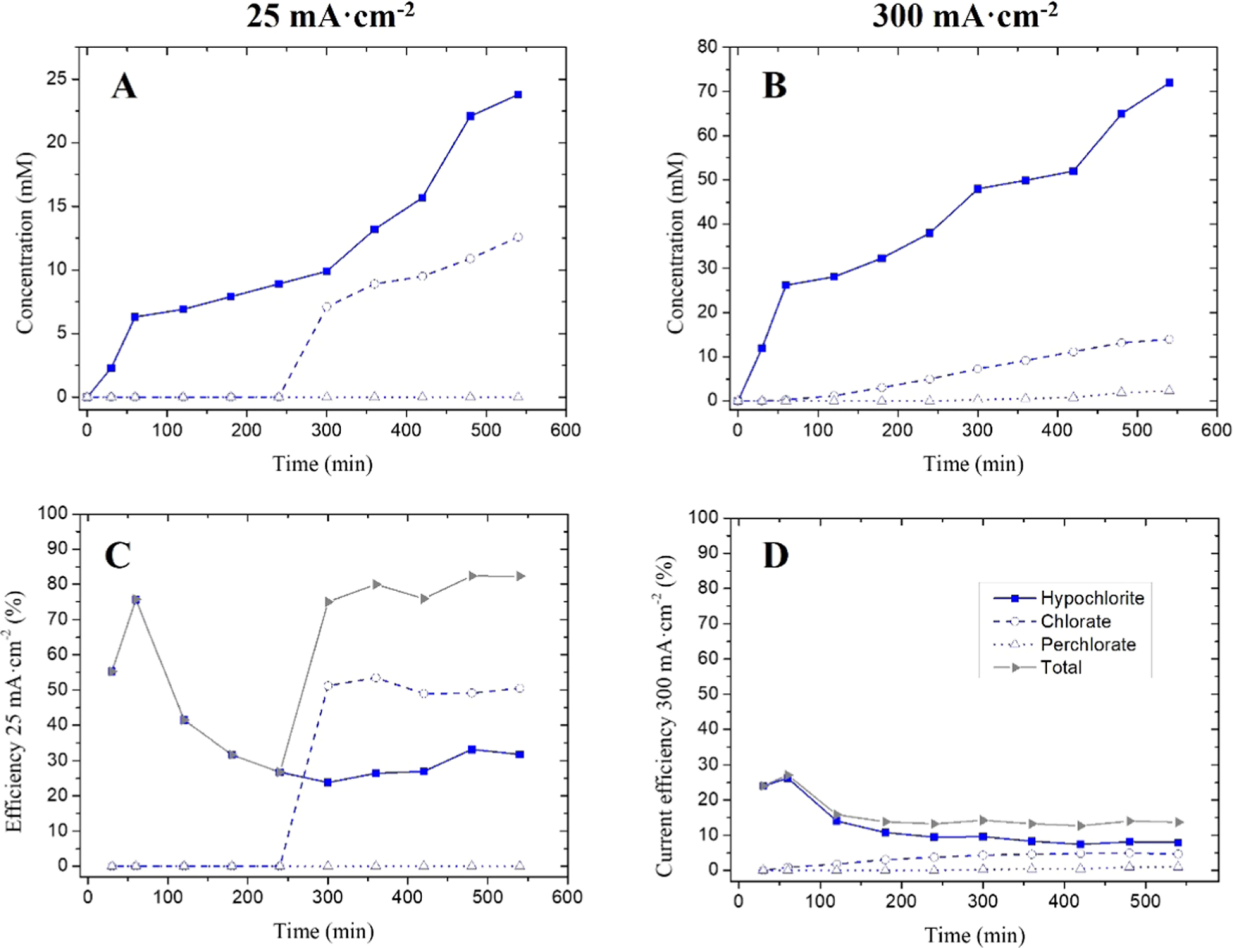
Oxidation chlorine products were obtained in a 0.5 M NaCl solution. Hypochlorite (blue bold line), chlorate (blue line with empty squares), and perchlorate (blue dotted line with empty triangles) are produced at 25 mA cm^–2^ (A) and 300 mA cm^–2^ (B) and their current efficiencies (C, D), respectively. The total current efficiency is represented in the gray line.

The production of oxidation products of chlorine species is represented in Figure 7. At 25 mA cm^–2^ (A), hypochlorite is the unique product observed until 300 min when chlorate starts to form. This behavior is reproduced at 300 mA cm^–2^ (B) but at an earlier time, at around 120 min. This may be because the hypochlorite produced is the species that is further oxidized to chlorate. The perchlorate is only observed at 300 mA cm^–2^ and at a long reaction time (420 min). This can be explained because perchlorate is formed by chlorate oxidation and a certain amount of chlorate is needed to start the formation of appreciable quantities of perchlorate. This behavior was also observed by Monteiro et al.^43^ Regarding the current efficiency, a steady 30% was obtained at 25 mA cm^–2^ (C) for hypochlorite production, and the value obtained for chlorate from 300 min was up to 50%. The combined efficiency of the process is up to 80% for over 500 min. When the current density is increased to 300 mA cm^–2^ (D), the current efficiency decays to 8, 5, and 1% for hypochlorite, chlorate, and perchlorate, respectively. The total efficiency achieved was 14% at 500 min. The data obtained indicate that the production of chlorine species at the BDD electrode is much more efficient at 25 mA cm^–2^. The efficiency value obtained is higher than those reported by Monteiro et al. for 25 mA cm^–2^, while for the higher current density of 300 mA cm^–2^, it is inferior. However, the electrode used is not the same in both studies.

## 4 Conclusions

A novel electrochemical cell (E3L-DAER) was designed to study boron-doped diamond electrodes. Several features including conic inlet/outlet parts, flow enhancers, and reduced interelectrode gaps, are proposed to reach near-ideal fluid dynamics conditions for BDD studies. The validation study included fluid dynamics and electrochemical parts. Several conclusions can be drawn.

- Other variations of the reactor without the proposed key features have been simulated by CFD and it has been demonstrated that both conic inlet/outlets and flow enhancers play a fundamental role in the flow distribution.
- The designed reactor fulfills the hydrodynamic conditions to perform reliable studies using BDD electrodes. These conditions include a fast pass of the liquid through the reactor, moderate mass transport, and optimal flow distribution through the entire electrode surface. In this sense, the recommended flow to operate the reactor is 50 L h^–1^.
- The E3L-DAER reactor was tested to produce different oxidant species and demonstrated to outperform the previous results obtained with the same BDD electrode, thus highlighting the importance of the reactor design in the production of oxidants.

## Supporting Information Available

Residence time distribution experiments (PDF)
