# Supplementary material for: Design, Validation, and Fabrication of a Tailored Electrochemical Reactor Using 3D Printing for Studies of Commercial Boron-Doped Diamond Electrodes
Source: Ind Eng Chem Res. 2024 Mar 25;63(13):5488–98. doi: 10.1021/acs.iecr.3c03123 (PMC10995994; doi:10.1021/acs.iecr.3c03123)
Supplement: Supplementary file 1 — ie3c03123_si_001.pdf [file ie3c03123_si_001.pdf]

Supporting Information

Design, validation, and fabrication of a tailored electrochemical reactor using 3D printing for studies of commercial boron-doped diamond electrodes

Lais Vernasqui[1,2], Miguel A. Montiel*[1], Neidenêi Gomes Ferreira[2], Pablo Cañizares[1], Manuel A. Rodrigo*[1]

1 Department of Chemical Engineering. Faculty of Chemical Sciences &Technologies. University of Castilla-La Mancha, Campus Universitario s/n, 13071 Ciudad Real, Spain

2 National Institute for Research Space. Av. dos Astronautas, 1.758 - Jardim da Granja, São José dos Campos - SP, 12227-010, Brazil

* Authors to who all correspondence should be addressed: Miguelangel.montiel@uclm.es

Manuel.rodrigo@uclm.es

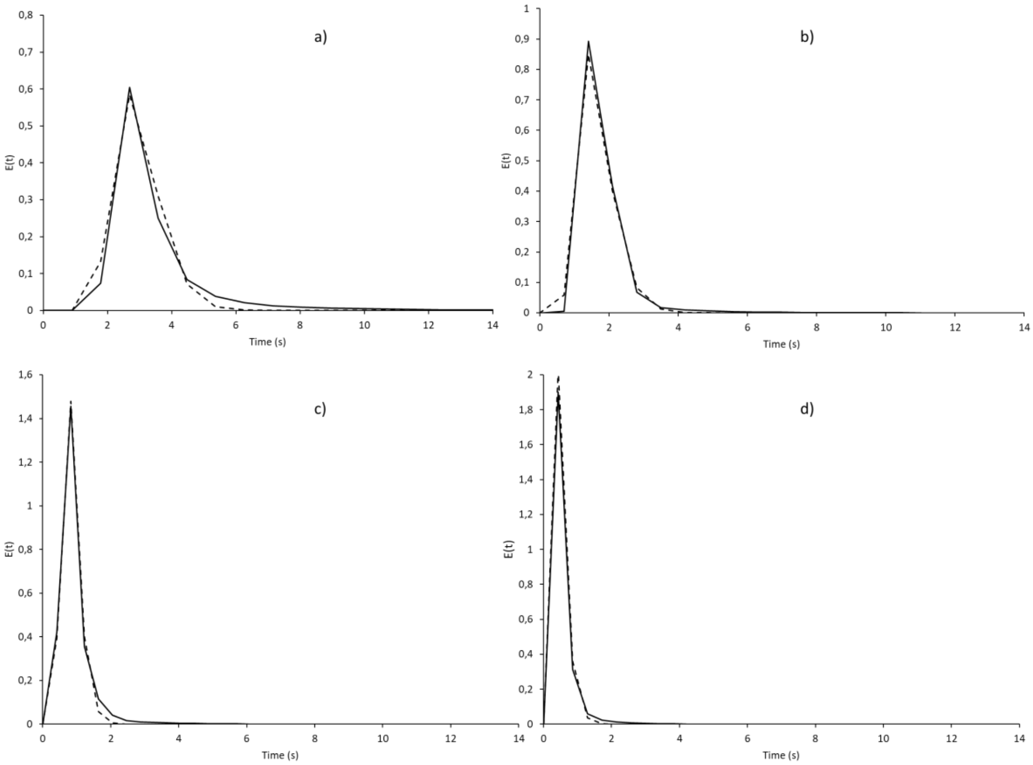

Figure S1. Residence time distribution experiment. NaCl trace is measured as conductivity at 15 (a), 30 (b), 50 (c), and 65 (d) $L \cdot h^{-1}$. E(t) experimental (solid line) and moddeled (dashed line) are represented.
